# Identification of SPP1 as an Extracellular Matrix Signature for Metastatic Castration-Resistant Prostate Cancer

**DOI:** 10.3389/fonc.2019.00924

**Published:** 2019-09-18

**Authors:** Xiaocong Pang, Ran Xie, Zhuo Zhang, Qianxin Liu, Shiliang Wu, Yimin Cui

**Affiliations:** ^1^Department of Pharmacy, Peking University First Hospital, Beijing, China; ^2^Department of Urology, Peking University First Hospital, Beijing, China

**Keywords:** SPP1, castration-resistant prostate cancer, extracellular matrix, metastasis, organoid model

## Abstract

Resistance to androgen deprivation therapy (ADT) is the main challenge for advanced fatal prostate cancer (PCa), which can gradually develop into metastatic castration-resistant prostate cancer (mCRPC). However, the pathologic mechanisms of mCRPC are still far from clear. Given the high incidence and mortality related to mCRPC, understanding the causes and pathogenesis of this condition as well as identifying potential biomarkers are of great importance. In the research reported here, we integrated several gene expression profiles from hormone sensitive prostate cancer (HSPC) and mCRPC datasets to identify differentially expressed genes (DEGs), key biological pathways, and cellular components. We found that extracellular matrix (ECM) genes were significantly enriched, and further filtered them using Pearson correlation analysis and stepwise regression to find ECM signatures to differentiate between the HSPC and mCRPC phenotypes. Six ECM signatures were input into K-nearest neighbor, logistic regression, naive Bayes, and random forest classifiers models. Random forest algorithm with the six-gene prognostic signatures showed best performance, which had high sensitivity and specificity for HSPC and mCRPC classification and further the six ECM signatures were validated in organoid models. Among the six ECM genes, SPP1 was identified as the key hub signature for PCa metastasis and drug resistance development; we found that both protein and mRNA expression levels of SPP1 were remarkably up-regulated in mCRPC compared with HSPC in organoid models and could regulate the androgen receptor signaling pathway. Therefore, SPP1 is a potential novel biomarker and therapeutic target for mCRPC. Further understanding of the role of SPP1 in mCRPC development may help to explore effectively therapeutic approaches for the prevention and intervention of drug resistance and metastasis.

## Introduction

Prostate cancer (PCa) is the most common malignancy and the second leading cause of death in males in the world (1). PCa is as an androgen-driven disease. Androgen deprivation therapy (ADT) is the mainstay of systemic treatment for locally advanced and metastatic prostate cancer (2). However, most patients initially response to ADT; however, they eventually develop from hormone sensitive prostate cancer (HSPC) to castration-resistant prostate cancer (CRPC) (3). Once PCa progresses to CRPC, tumor cells gradually develop an invasive phenotype with significant potential for invasion and metastasis (4). The high mortality of PCa is mainly attributable to tumor metastasis and drug resistance, but the mechanisms associated with progression are largely unknown, and there is an urgent need for the identification of biomarkers for the development of metastatic CRPC (mCRPC).

Numerous efforts have been made to identify signatures of the tumor epithelial components in some retrospective studies, but few biomarkers are currently considered adequate to establish the diagnosis and prognosis of prostate cancer (5, 6). Polyclonal characteristics of tumor heterogeneity and cumulative genetic alterations provide a significant challenge for biomarker discovery (7, 8). Recently, elements of the extracellular matrix (ECM) have been proposed as biomarkers for the progression and metastasis prognosis for several types of tumor (9, 10). The interaction between tumor cells and ECM components promotes tumor cells invasion and metastasis (11). Penet et al. found the structure and function of ECM could facilitate PCa metastasis (12). In this prospective study, Lucarelli et al. also found SPON2, a secreted ECM protein, is a potential biomarker for prostate cancer diagnosis as well as has good association with clinicopathological features (13).

Recently, studies have shown that interactions between tumor cells and ECM constituents are central to key aspects of drug resistance, especially in solid tumor (14, 15). For example, in bone metastasis CRPC, many integrins, transforming growth factor-beta (TGFβ) family members, bone resident proteins, nuclear factor kappa B receptor activating factor ligand (RANKL), and parathyroid hormone related proteins (PTHrP) are involved in matrix remodeling, which can lead to drug resistance (16, 17). The results of numerous investigations suggest that there are many changes in gene expression at the RNA and protein levels, which are specific to the tumor microenvironment of prostate cancer (18, 19). However, for advanced PCa, the association between ECM gene changes and the occurrence of mCRPC has not yet been studied in depth (20, 21), and investigating the role of ECM components in drug resistance and illustrating the cross talk between tumor cells and microenvironment niches would benefit the development of new therapeutic strategies.

Herein, to identify the ECM signatures during the development of mCRPC, we investigated the gene expression profiles of HSPC and mCRPC samples and integrated a variety of benchmark datasets. Through analyzing differentially expressed genes (DEGs), key biological pathways, and cellular components, ECM genes were significantly enriched, and then K-nearest neighbor, logistic regression, naive Bayes, and random forest classifiers were employed to identify ECM signatures which could distinguish between HSPC and mCRPC phenotypes. Finally, organoid models were built to validate the results *in vitro*. This study made a substantial contribution to our understanding of the roles of ECM CRPC progression, and helps to the discovery of new potential biomarkers and treatment targets.

## Materials and Methods

### Gene Expression Data and Processing

Gene expression profiles GSE32269, GSE101607, and GSE3325 of mCRPC and PCa tissue were obtained from GEO database, a public functional genomics data repository. GSE32269 series consists of 22 primary PCa (hormone-dependent) and 29 mCRPC acquired using the GPL96 platform (Affymetrix Human Genome U133A Array). GSE101607 compares eight HSPC vs. 40 mCRPC acquired using the GPL10558 platform (Illumina HumanHT-12 V4.0 expression beadchip). There are five primary PCa and three mCRPC in GSE3325, which were produced using the GPL570 platform (Affymetrix Human Genome U133 Plus 2.0 Array). We also downloaded GSE74685, which contains data pertaining to 149 visceral and bone metastases from 62 mCRPC patients.

We integrated and processed the GSE32269, GSE101607, and GSE3325 data sets using the sva package in the R language to reduce the bias and variability inherent in different sequencing results. The sva package supports the use of sva functions for proxy variable estimation, the ComBat function to directly adjust known batch effects, and the fsva function to adjust batch and latent variables in predictive problems. Class comparison analysis for DEGs was conducted with the limma Bioconductor package (R version 3.5.1) and DEGs with *p* < 0.05 and |log_2_FC| >1 were selected for further analysis. Kolmogorov Smirnov test was used for comparing the expression of genes in different metastatic sites in the mCRPC samples from GSE74685 data set.

### Gene Ontology (GO) and KEGG Pathway Enrichment

KEGG pathway enrichment was analyzed using Clusterprofiler, an R package with analysis and visualization. The Database for Annotation, Visualization and Integrated Discovery (DAVID, https://david.ncifcrf.gov/) (22) was used for GO biological pathway and cellular component enrichment. The GOplot package was used to combine and integrate expression data with the results of the DAVID analysis. Membrane molecules annotated to the ECM by GO cellular component (GO_CC) were used to construct a protein-protein interaction network using data from the STRING database. The network of interactions was visualized using Cytoscape 3.5.1 (23). Gene Set Enrichment Analysis (GSEA) (24) was also employed to find biological function gene sets regulated by the hub gene in the network. Based on the median expression of hub gene (25), all the datasets were divided into two groups (high expression vs. low expression). GSEA v2.0 (http://software.broadinstitute.org/gsea/downloads.jsp) was used with the parameter of number of permutations set at 5, and the threshold pf enrichment result was *P* < 0.05.

### Classification by Machine Learning

Pearson correlation analysis was applied to eliminate low correlation and high-auto-correlation between phenotypes and signatures. The phenotypes of HSPC and mCRPC were indicated as “0” and “1,” respectively. Genes with a correlation coefficient <0.1 were removed, and if the coefficient between two phenotypes was beyond 0.9, the gene with a lower correlation coefficient was also deleted. The remaining genes with *p* < 0.05 were further analyzed by a stepwise regression approach, which considered variable size, significance, and contribution. Finally, the regression equation was established by considering signatures one by one. Every new regression equation was subjected to a significance test to evaluate the addition of each new signature. The process terminated when there were no new signatures imported or deleted. Using the selected gene signatures, we used K-nearest neighbor (KNN), logistic regression (LR), naive Bayes (NB), and random forest (RF) algorithms to construct HSPC and mCRPC classification models, using Orange Canvas 3.13 (26). The performance of models was estimated by 5-fold, random sampling and leave-one-out cross-validation.

### Organoid Development and Culture

Biopsy tissues were obtained from patients with advanced prostate cancer after ethical approval. The tissues were washed with cold PBS containing antibiotics and chopped into small pieces with surgical scissors. Tissues were further washed with 10 mL AdvancedDMEM/F12 and digested in 10 mL AdvancedDMEM/F12 containing 2% FCS and 2 mg/ml collagenase (Sigma, C9407) on an orbital shaker at 37°C for 0.5–1 h. The pellet was resuspended in 10 ml Advanced DMEM/F12 containing 2% FCS and centrifuged again at 400 rcf. Dissociated cells were collected in Advanced DMEM/F12 (Thermo Fisher Scientific, Waltham, MA, USA), suspended in growth factor reduced (GFR) matrigel (Corning Inc., Corning, NY, USA), and seeded. The matrigel was then solidified and overlaid with 500 μl of complete human organoid medium, which was subsequently refreshed every two days. PDOs were cultured in Advanced DMEM/F12, supplemented with 1x B27 additive and 1x N2 additive (Thermo Fisher Scientific, Waltham, MA, USA), 0.01% bovine serum albumine, 2 mM L-glutamine, 100 units/ml penicillin-streptomycin, and containing the following additives: EGF, noggin, R-spondin 1, gastrin, FGF-10, FGFF-basic, Wnt-3A, prostaglandin E2, Y-27632, nicotinamide, A83-01, SB202190, HGF (Pepro-Tech, London, UK). Passaging of PDOs was performed using TrypLe. PDOs were biobanked in FBS (Thermo Fisher Scientific, Waltham, MA, USA), containing 10% DMSO (Sigma- Aldrich, St. Louis, MI, USA). In this study, we used three HSPC (KOPCa-030,031,032) and three mCRPC (KOPCa-001,012,017) organoid models. Organoid models were evaluated with inverted microscope and hematoxylin-and-eosin (H&E) staining. The inverted microscope was used for bright field image analysis. Prostate cancer organoids were stained with hematoxylin-and-eosin (H&E) for analyzing the histomorphometry. The organoid was cultured with prostate-specific medium, and the ingredients of culture media were reported in previous study (27). The organoids were cultured in a cell culture incubator at 37°C and 5% CO_2_. The culture was replenished with fresh media every 3–4 days during organoid growth.

### Real-Time Quantitative PCR (RT-qPCR)

The mRNA expression levels of ECM genes, hPSMA, hAR, and hPSA in human prostate organoids were detected using RT-qPCR. The qRT-PCR protocol has been described in previous publications (28). One hundred organoids (~1 × 10^6^ cells) were collected and total RNA was extracted from cell pellets using Easy pure RNA Kits (TransGen Biotech, BJ, China). RNA was performed reverse transcription using TransScript First-Strand cDNA Synthesis SuperMix (TransGen Biotech, BJ, China) following the manufacturer's protocol. After an initial hot start at 95°C for 10 min, PCR amplification was carried out for 40 cycles, denaturation at 95°C for 15 s, and annealing and extension at 60°C for 1 min. The relative levels of gene expression were detected by the cyclic threshold method.

### Immunohistochemical Staining

Immunohistochemical staining was utilized to determined SPP1 expression in human prostate organoids. The details of organoid staining procedures were similar to the description in previous study (29). After dewaxing in xylene and rehydration in ethanol, the sections were immersed in methanol containing 3% hydrogen peroxide, then rinsed in tap water and immersed in distilled water. Slides were incubated with anti-SPPl antibody (ab8448, Abeam, MA) in PBS at 1:800 dilution overnight at 4°C and then treated with a secondary antibody. Sections were stained with hematoxylin, dehydrated and fixed, and sections lacking primary antibodies were used as negative controls.

### Statistical Analysis

All data in this study were showed as means ± standard error. Student's *t*-test was used to examine the statistical significance between HSPC and mCRPC groups, and the significant *p*-value was considered to be lower than 0.05.

## Results

### Screening of DEGs

**Figure 1:**
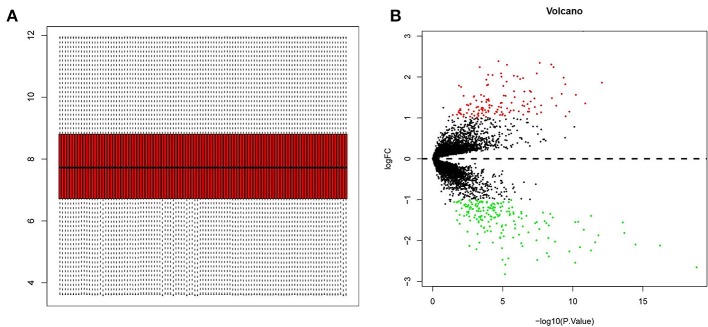
Screening of differentially expressed genes (DEGs) in mCRPC tissues compared with HSPC tissues. **(A)** GSE32269, GSE101607, and GSE3325 datasets (35 PCa and 72 mCRPC samples) were merged and normalized by sva package in R language. **(B)** Volcano plots of all quantified genes in the gene expression profiles analysis of mCRPC and HSPC. Volcano map identified statistically significant DEGs (*p* < 0.05 and |log_2_FC| > 1), and totally there were 130 up-regulated DEGs (red dot) and 184 down-regulated DEGs (green dot).

The GSE32269, GSE101607, and GSE3325 data sets, totally including 35 HSPC and 72 mCRPC samples, were merged and processed using the sva package in R. Totally, 8,364 genes were analyzed and normalized (Figure 1A). The limma Bioconductor package was employed to analyze the DEGs in the integrated data sets. After filtration with a threshold of *p* < 0.05 and |log_2_FC| > 1, there were 130 up-regulated DEGs and 184 down-regulated DEGs in mCRPC tissues compared with HSPC tissues (Supplement 1). The DEGs are shown in the volcano map (Figure 1B) and their *P*-value and log_2_FC value were listed in the supplements.

### GO and Pathway Enrichment Analysis

**Figure 2:**
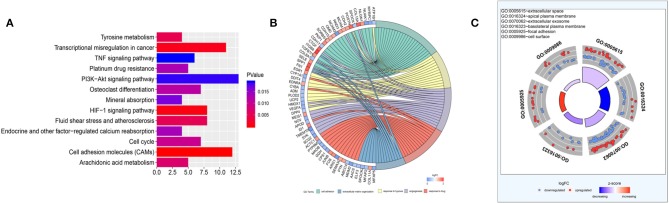
Functional enrichment analysis. **(A)** KEGG analyses of the pathways of DEGs. The x axis shows the gene numbers and the color of bar represents the *p*-value. There were 13 pathways meeting the cutoff value of *p* < 0.05. **(B)** Gene Ontology analyses of DEGs according to their biological process. ECM genes, likes POSTN, COL8A1, SPP1, and FN1 were the key intersectional points of the GO_BP terms. **(C)** GO cellular component enrichment analysis.

**Figure 3:**
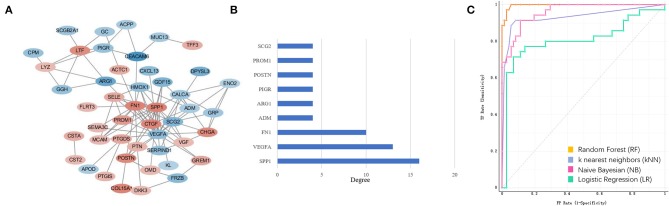
Extracellular matrix (ECM) gene interaction and selection. **(A)** ECM genes interaction network built using STRING The red circles represent up-regulated genes and the blue circles represent down-regulated genes. The fold-change values of DEGs present with different color shades. **(B)** The degree of the ECM gene with count of node more than four. **(C)** ECM signatures identified by machine learning methods during mCRPC development. ROC curves of four PCa and mCRPC classification models.

All the DEGs, totally 314 genes, were employed as input for GO and KEGG enrichment analysis. The Clusterprofiler R package was applied to analyze the enrichment of KEGG pathways. There were 13 KEGG pathways (Figure 2A) meeting the cutoff value of *p* < 0.05, including cell adhesion molecules (CAMs), the HIF-1 signaling pathway, transcriptional misregulation in cancer, tyrosine metabolism, fluid shear stress and atherosclerosis, arachidonic acid metabolism, cell cycle, osteoclast differentiation, platinum drug resistance, endocrine and other factor-regulated calcium reabsorption, mineral absorption, PI3K-Akt signaling pathway, and the TNF signaling pathway. Among them, CAMs had the highest significance and the PI3K-Akt signaling pathway was enriched with the most DEGs. GO biological pathway (GO_BP) enrichment was analyzed using the DAVID database and its tools (Figure 2B). Cell adhesion, ECM organization, response to hypoxia, angiogenesis, and response to drug were the top GO_BP terms having high significance and including 10 or more genes. ECM genes, likes POSTN, COL8A1, SPP1, and FN1 were the key intersectional points of the GO_BP terms. GO_CC enrichment (Figure 2C) was used to summarize the main cellular component in the mCRPC progression. According to enrichment results, there were 53 ECM genes (enriched in extracellular space or extracellular exosome) significantly differently expressed in mCRPC compared with HSPC samples. Based on the analysis of the interactions of ECM genes conducted using STRING (Figure 3A), SPP1 was identified as the key hub gene in the network with the highest degree (Figure 3B).

### *In silico* Analysis of ECM Genes

To explore the ECM signatures modulated during mCRPC development, HSPC and mCRPC classifiers were built using machine learning algorithms. Before developing the models, Pearson correlation analysis were applied to filter the 53 ECM genes (Supplement 2). There were 39 genes with *p* < 0.05, which were used as the input variables for stepwise regression. Stepwise regression basically performs multiple regressions, removing the weakest relevant variables each time. After stepwise regression, six variables were left, which can illustrate the distribution best, including SPP1, CEACAM6, COL15A1, FN1, POSTN, and ARG1. The six genes were used as ECM signatures to build NB, KNN, LR, and RF classification models. Table 1 shows the performance of the four models, as estimated using 5-fold, random sampling and leave-one-out cross-validation. The RF classification model showed the best performance, and both of precise and AUC of the three evaluation methods were beyond 0.915. ROC analysis also showed that the RF algorithm with the six-gene prognostic signature had high sensitivity and specificity for PCa and mCRPC classification (Figure 3C).

**Table 1:** Performance of Naive Bayes (NB), K-nearest Neighbor (KNN), Logistic Regression (LR) and Random Forest (RF) models estimated by 5-fold, random sampling, and leave-one-out cross-validation.

**Classification model**	**5-fold cross validation**		**Random sampling**		**Leave one out**	
	**Precise**	**AUC**	**Precise**	**AUC**	**Precise**	**AUC**
Logistic Regression (LR)	0.842	0.849	0.846	0.786	0.842	0.741
Random Forest (RF)	0.954	0.995	0.915	0.989	0.954	0.953
Naive Bayesian (NB)	0.889	0.948	0.896	0.799	0.880	0.925
K-Nearest Neighbors (kNN)	0.925	0.927	0.874	0.895	0.925	0.879

### Validation in Patient Derived Organoids (PDTO) Model

**Figure 4:**
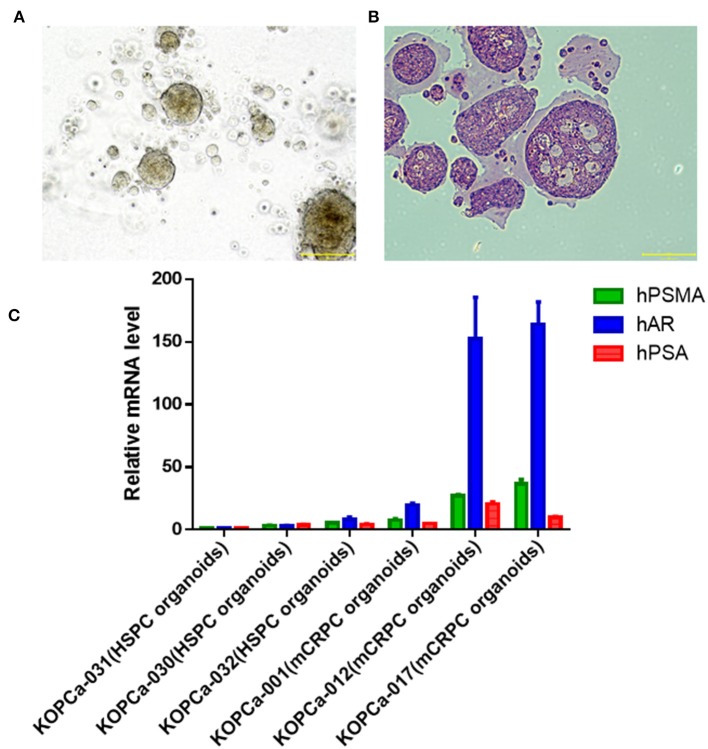
Development of patient-derived prostate cancer models. **(A)** Bright field image analysis (magnification X100, scale bar 200 μm) of prostate cancer organoid (KOPCa-031). **(B)** Prostate cancer organoid (KOPCa-031) is stained with hematoxylin-and-eosin (H&E) (magnification X100, scale bar 200 μm). **(C)** Molecular characterization of patient-derived prostate cancer models through evaluating the mRNA expression of hPSMA, hAR, and hPSA by real-time PCR. All the prostate cancer organoid models [three HSPC (KOPCa-30,31,32) and three mCRPC (KOPCa-001,012,017)] could express the prostate cancer-specific molecular markers.

**Figure 5:**
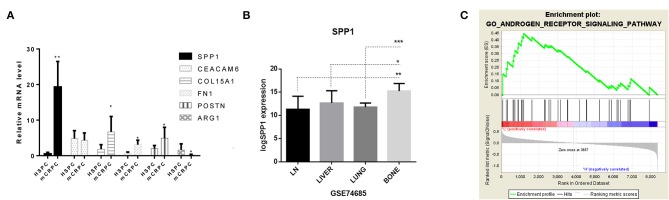
Identification of the role of SPP1 in mCRPC. **(A)** Real-time PCR results of SPP1, CEACAM6, COL15A1, FN1, POSTN and ARG1 mRNA expression in HSPC and mCRPC organoids. **(B)** Expression of SPP1 in different metastatic sites in GSE74685, including the lymph nodes, liver, lung, and bone. *P* values were obtained by two-tailed unpaired Student's *t*-tests, *p < 0.05, **p < 0.01, and ***p < 0.001. **(C)** Gene Set Enrichment Analysis (GSEA) of SPP1 regulated pathways. According to the expression of SPP1 in GSE32269, GSE101607, and GSE3325 data sets (35 HSPC and 72 mCRPC samples), the data sets were divided into SPP1 low-expressed datasets and SPP1 high-expressed datasets. Single-gene GSEA was analyzed to identify the pathways regulated by SPP1. The androgen receptor signaling pathway is significantly enriched.

The PDTO model technique is a cutting-edge technology for *in vitro* three-dimensional culture of tumor precision medicine. The PDTO model can replicate the tissue complexity and genetic heterogeneity of tumors, showing good potential in modeling success rate, maintenance difficulty and screening difficulty (30). Fresh tumor tissues from six patients with prostate cancer were successfully used for organoid development. From the bright field image analysis and H&E staining results (Figures 4A,B), the organoid model could retain certain tumor tissue pathological information. The tumor cells in the 3D cultured organ *in vitro* formed a compact spherical structure. H&E staining showed that the epithelioid-like structure formed by the organoid model. It was found that the molecular markers (hPSMA, hAR, and hPSA) were detected from three HSPC (KOPCa-030,031,032) and three mCRPC (KOPCa-001,012,017) in Figure 4C. To investigate the prognostic prospects of the six ECM gene, we further examined the expression of SPP1, CEACAM6, COL15A1, FN1, POSTN and ARG1 in HSPC, and mCRPC organoids using RT-qPCR. Among the six signatures, SPP1 had the most significant variation. Figure 5A shows that high levels of SPP1 mRNA were expressed in mCRPC (*p* < 0.01). COL15A1, FN1, and POSTN were also highly expressed in mCRPC with *p* < 0.05, which was also consistent with the GEO expression profiles analysis. ARG expression was significantly down-regulated in mCRPC organoids (*p* < 0.05), but CEACAM6 expression was not significantly changed. Carcinoembryonic antigen-related cell adhesion molecule 6 (CEACAM6) is a member of the immunoglobulin supergene family, and its expression is elevated many solid tumor, but variable as a function of histotype (31, 32). Herein, the expression of CEACAM6 was not significantly dysregulated in mCRPC organoids.

### Role of SPP1 in Promoting mCRPC Progression

**Figure 6:**
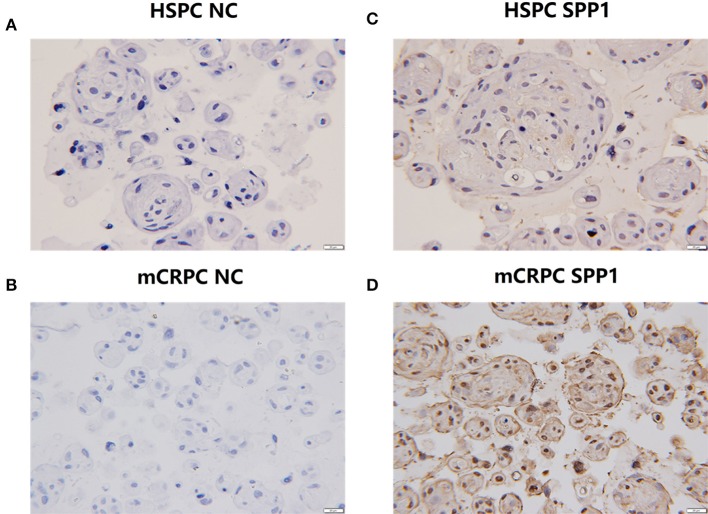
IHC analysis of SPP1 in HSPC and mCRPC organoid samples. **(A,B)** The negative controls for HSPC and mCRPC, respectively. **(C,D)** Expression of SPP1 in mCRPC organoids is significantly lower than that of HSPC organoids. Original magnification 100X.

According to the bioinformatics analysis described above, SPP1 was the key hub ECM gene for the development of mCRPC, and SPP1 mRNA expression was also most significantly dysregulated among the six ECM signatures. SPP1, also known as Osteopontin (OPN), is an important secretory phosphorylated glycoprotein producted by both tumor cells and a variety of host cells, including multiple immune cells, osteoclasts, fibroblasts, endothelial, smooth muscle and epithelial cells (25). SPP1-induced interaction between tumor cells and stromal cells promotes tumor progression and angiogenesis, and drug resistance. However, there are rare studies of OPN mediated drug resistance of prostate cancer, and the role of SPP1 in mCRPC is still elucidated. Bone metastasis frequently occurs in majority of patients with PCa, which lead to devastating complications due to skeletal-related events (SREs) and reducing patients' survival. Expression of SPP1 varied in different metastatic sites (Figure 5B), and especially high expression of SPP1 was observed in bone metastasis. Actually, SPP1 could promote bone metastasis progression by stimulating immune cells, activating endothelial cells and fibroblasts, and regulating osteoclasts and osteoblasts mediated formation and resorption in the bone microenvironment (33). According to the expression of SPP1 in GSE32269, GSE101607, and GSE3325 data sets (35 HSPC and 72 mCRPC samples), the data sets were divided into SPP1 low-expressed datasets and SPP1 high-expressed datasets. Based on GSEA results, SPP1 could significantly regulated androgen receptor signaling pathway in high-expression group, which has been considered as the main driver of CRPC progression (Figure 5C). Figure 6 showed the results of IHC for HSPC and mCRPC organoids with the SPP1-specific monoclonal antibody. Weak staining of SPP1 was observed in PCa organoids (Figures 6A,B), while the intensity of staining in mCRPC organoids was significantly stronger than in PCa organoids (Figures 6C,D). Therefore, SPP1 mRNA and protein levels were highly elevated in mCRPC organoids.

## Discussion

In this study, we integrated three GEO datasets and performed bioinformatics analysis using several machine learning algorithms to find signatures related to mCRPC progression. By performing GO and pathway enrichment analysis, we found that cell adhesion, extracellular matrix organization, and drug resistance-related pathways were significantly dysregulated and ECM genes appeared to be related to mCRPC development (Figures 2, 3).

The dysfunction of CAMs is a hallmark of the epithelial-to-mesenchymal transition, leading to the aggressive phenotype transformation of tumor cells, such as that exhibiting stem cell-like features and treatment resistance (34). The CAMs are mainly cadherins and integrins. CDH2 (cadherin 2, also known as N-cadherin) is a transmembrane glycoprotein, which was significantly up-regulated in the mCRPC group. CDH2 could mediate calcium-dependent cell adhesion and promote prostate cancer cells metastatic activity, and its aberrant expression has been reported in both metastatic cancer and CRPC (35). It is well known that the PI3K/Akt pathway regulates tumor cell progression, adhesion, invasion and survival (36, 37). In addition, the crosstalk between PI3K-Akt signaling pathway and androgen receptor (AR) signaling axis plays an important role in the CRPC progression (38).

Six ECM-derived signatures were identified and used to build PCa and mCRPC classification models based on four machine learning algorithms, including NB, KNN, LR, and RF, which were further validated through 5-fold, random sampling, and leave-one-out cross-validation. ECM-derived signatures included SPP1, CEACAM6, COL15A1, FN1, POSTN, and ARG1, which mRNA expression was validated in organoid models. PCa tissue specimens obtained from patient biopsy, were performed organoid culture, and rapidly realize tissue culture and amplification. Figure 4 showed the HSPC and mCRPC organoid models preserved the histopathology and molecular characteristics of PCa. According to RT-qPCR results, among the six ECM signatures, only the expression of CEACAM6 was not significantly changed. CEACAM6 is overexpressed in many solid tumors, including pancreatic, colon, breast, and lung cancer, but its expression is variable in different histological phenotypes of metastases (32). The mRNA expression of SPP1, COL15A1, FN1, POSTN, and ARG1 were significantly dysregulated in mCRPC organoid models compared with HSPC organoid models. In the basement membrane zones, COL15A1 is the highest expressed COL and its expression has a strong relationship with drug resistance in ovarian cancer cell lines (39). FN1 (fibronectin 1) is the most important member of the fibronectin family, which is essential ECM glycoprotein, regulating tumor cell adhesion and migration. Fibronectin can up-regulate matrix metalloproteinases (MMPs) expression in human prostate cell lines (40). High POSTN expression was found to be correlated with aggressive tumor behavior, advanced or poor prognosis, indicating that POSTN is a useful biomarker for advanced cancer (41). ARG1, which encodes Arginase1, is involved in anti-inflammation, tumor immunity, and immunosuppression-related diseases (42).

The data mining results showed that SPP1 expression was highly elevated in mCRPC, and network analysis of ECM interactions also revealed SPP1 to be the central hub gene (Figure 3A). The RT-qPCR results also suggested SPP1 mRNA levels had the most significantly changed, and immunohistochemical staining was further performed to determine SPP1 protein levels. The protein and mRNA levels of SPP1 were significantly increased in mCRPC compared with HSPC (Figure 6), in addition, SPP1 expression varied in different metastatic site (Figure 5B). SPP1 is a secreted chemokine-like glycophosphoprotein participated in tumor cell proliferation, invasion, and metastasis (43). Clinically, SPP1 expression level in tumor tissue and plasma is associated with poor prognosis and survival in patients with PCa (44, 45). It also suggested that SPP1 could upregulated the expression p-glycoprotein (P-gp), which mediates multidrug resistance in PCa cells (46). In a randomized phase III trial, SPP1 combined with other serum biomarkers, such as hepatocyte growth factor and C-peptide can predict the prognosis of ADT in metastatic androgen-dependent prostate cancer (mADPC) patients (47). However, the role of SPP1 in the ADT-induced acceleration of mCRPC still need further study. According to the GSEA result with respect to the SPP1 regulated pathways, the AR signaling pathway was significantly enriched. Therefore, SPP1 is a potential biomarker and action target for mCRPC diagnosis and treatment. Decreasing of SPP1 at the transcriptional or protein level, or inhibiting SPP1 binding to integrin avβ3 and CD44 receptor or the downstream pathways, was able to be effective therapeutic methods for mCRPC.

In conclusion, different series from the GEO database were integrated and processed to investigate the pathogenesis of mCRPC, and six ECM signatures were identified as capable of distinguishing mCRPC from HSPC with RF classification models. In addition, combining the validation results in organoid models, we found SPP1 was the potential hub signature for predicting mCRPC progression for the first time. Further understanding of the role of SPP1 in mCRPC could help in the development of effective therapeutic approaches for the prevention and intervention of metastasis and drug resistance in advanced PCa.

## Data Availability

The available public datasets from GEO database (https://www.ncbi.nlm.nih.gov/gds) were downloaded and analyzed in this study. The data analyzed in this study could be obtained from the Supplementary Files.

## Author Contributions

XP and YC conception and design and obtained funding. XP conducted the analysis and interpretation of the data, and drafted the manuscript. RX acquired the data and drafted the manuscript. ZZ and QL statistical analysis and technical support. SW critically revised the article for important intellectual content.

### Conflict of Interest Statement

The authors declare that the research was conducted in the absence of any commercial or financial relationships that could be construed as a potential conflict of interest.
